# Demystifying physiologically based pharmacokinetic modelling among non-modelers towards model-informed medicine use in under-served populations.

**DOI:** 10.12688/gatesopenres.14896.1

**Published:** 2023-10-26

**Authors:** Jolien Freriksen, Joyce van der Heijden, Marika de Hoop-Sommen, Trevor Johnson, Karen R Yeo, Essam Kerwash, Susan Cole, Janet Nooney, Rick Greupink, Ping Zhao, Saskia de Wildt

**Affiliations:** 1 Radboud University, Nijmegen, Gelderland, The Netherlands; 2 Certara UK Limited, Sheffield, UK; 3 Medicines and Healthcare Products Regulatory Agency, London, UK; 4 Bill & Melinda Gates Foundation, Seattle, Washington, USA; 5 Erasmus MC-Sophia Children's Hospital, Rotterdam, The Netherlands

**Keywords:** Dosing, Under-served Populations, Model-informed Drug Development, Pediatrics, Pharmacokinetics, Pregnancy

## Abstract

Physiologically based pharmacokinetic (PBPK) models represent computational technology to characterize drug behavior within the context of detailed human physiology. Today, PBPK is routinely used in drug development and regulatory approval to support decisions on how a medicine can be used under certain clinical conditions. As such, PBPK has the potential to enhance medicine use for populations that are often under-served globally in drug development and clinical care, namely pediatric patients, pregnant and lactating women. To facilitate broader applications of PBPK for these populations, we joined force and organized five hands-on workshops primarily to non-modelers on the principles of PBPK and its potential applications in pediatric and obstetric pharmacology in 2021 and 2022. In this open letter, we report learning objectives and content of such workshops and to highlight the significant value of these educational efforts.

## Disclaimer

The views expressed in this article are those of the author(s). Publication in Gates Open Research does not imply endorsement by the Bill & Melinda Gates Foundation. The views may not be understood or quoted as being made on behalf of or reflecting the position of the Medicines and Healthcare products Regulatory Agency, UK.

## Ethics statement

As it is not a medical/health research, no otherwise sensitive information was collected, participation was voluntarily and data were processed anonymously, explicit written informed consent was not sought.

## Value of physiologically based pharmacokinetic modeling for under-served populations

There is a general lack of on-label drug dosing information for pediatric patients, pregnant and lactating women ^1, 2^. Historically, these populations have hardly been evaluated in clinical programs during the development of drug products. Even when clinical studies are conducted, the generalizability of such studies can be limited because the effect of relevant age and/or pregnancy-related physiological and anatomical variations can significantly complicate a drug’s pharmacology in a specific subgroup of these populations, calling for different dosage adjustments in different subgroups. To this end, computational tools such as physiologically based pharmacokinetic (PBPK) modeling which characterize the physiological changes can be used to support dosing decisions ^3– 5^.

A PBPK model consists of several compartments representing organs and tissues. Maturational effects of physiology can be described using (gestational) age-dependent equations. This allows the creation of a set of virtual subjects, each representing anatomical and physiological characteristics of pediatric subjects or pregnant and lactating women at a specific developmental stage (the system model). In parallel, one can compile drug-dependent parameters to construct a drug model, and couple it with the system model through relevant physiological processes concerning the absorption and disposition of the drug. A user-defined virtual trial then can be conducted to simulate pharmacokinetic (PK) characteristics in a specific virtual subject or sub-population. Such PK information can then form the basis for dosing decisions ^6^.

In the last two decades, advancements in computation and pharmacological sciences fueled the industrialization of PBPK. Today, PBPK is a platform technology used routinely by drug developers and regulatory agencies to inform dosing recommendations for a wide range of applications ^7^. Interest in PBPK modeling to predict PK in pediatric and pregnant subjects has increased dramatically over the past few years ^6, 8^. Of all regulatory submissions involving PBPK modeling received by the US Food and Drug Administration (FDA) in 2018 – 2019, 9% were related to pediatric population ^7^. Application of pediatric PBPK models also increased in clinical and research settings, for both biologics and small molecules ^9^. While *in silico* PBPK modeling is generally conducted to inform clinical trial design in pediatric and pregnant populations, it can also be applied in the post marketing stage to inform drug dosing ^10^.

## Demystifying PBPK among non-modelers towards broader application in under-served populations

PBPK is ideal to investigate and predict effects of dynamic physiological changes on a drug’s pharmacology. Although the FDA reported emerging use of PBPK in pediatrics, no application was recorded to inform drug dosing in pregnant and lactating women ^7^. This gap can be attributed to the low confidence in PBPK for these intended uses, and/or the lack of understanding of today’s PBPK as a platform technology by broader medical communities. While the former can be continuously improved through increased PBPK research in the space of pediatric and obstetric pharmacology ^6, 8^, the latter requires introduction of PBPK to stakeholders who have little or no experience in pharmacology modeling. As such, we joined force and conducted five workshops in 2021 and 2022 primarily targeting these non-modeler audiences ( Table 1). 

**Table 1.** Five PBPK modeling workshops held in 2021 – 2022. ^a^Radboud Summer School of 2021 also included exercises related to applying PBPK modeling to evaluate in vitro-to-in vivo extrapolation and drug-drug interactions in healthy subjects. ^b^ MHRA workshop aimed at those primarily interested in PK changes in pregnancy, rather than PBPK modeling specifically. (Supplemental file 1)

Workshop	Date, location and duration	Attendees	Populations discussed	Number (% total) attendees participated in survey before the workshop	Number (% total) attendees participated in survey after the workshop
Part of the Radboud Summer School “Introduction to Pharmacokinetic and Pharmacodynamic Analysis”	July 2021 Online Half day	26 delegates ^a^ (global) 5 tutors	Pediatric and pregnant population	22 (85%)	12 (46%)
Pre-congress course of the 19th European Society for Developmental Perinatal and Paediatric Pharmacology (ESDPPP) meeting	June 2022 In-person Liverpool (UK) One day	37 delegates (mostly European) 11 tutors	Pediatric and pregnant population	18 (49%)	9 (24%)
Part of the Radboud Summer School “Introduction to Pharmacokinetic and Pharmacodynamic Analysis”	July 2022 Online Half day	29 delegates ^a^ (global) 5 tutors	Pediatric and pregnant population	28 (97%)	21 (72%)
Satellite Session of the American Society for Clinical Pharmacology & Therapeutics (ASCPT) meeting	September 2022 Online One day	45 delegates (global) 14 tutors	Pediatric and pregnant population	33 (73%)	12 (27%)
Medicines and Healthcare products Regulatory Agency (MHRA) workshop	October 2022 In-person London (UK) One day + two (1hr) online pre-event sessions	32 delegates ^b^ (mostly UK) 7 tutors	Pregnant population	Not applicable as all feedback was collected after the workshop	20 (63%

These workshops target clinicians, pharmacists, academic researchers and scientists from non-governmental organizations, ranging from the (PhD) student to senior fellows, as well as clinical assessors from regulatory agencies, with an interest in pediatrics and/or obstetrics pharmacology. Recruitment of participants were by targeted advertising and selection for MHRA workshop, and through announcement by Radboud Summer School, European Society for Developmental Perinatal and Paediatric Pharmacology (ESDPPP), and American Society for Clinical Pharmacology & Therapeutics (ASCPT). No inclusion/exclusion criterion was specified. No prior knowledge of PBPK modeling and accessibility to PBPK modeling software was required for Radboud Summer School, ESDPPP, and ASCPT events.

All workshops shared similar features by including theoretical and hands-on sessions. The theoretical sessions were designed to

(a) Disseminate the basics of PK and PBPK modeling.
(b) Demonstrate the utility and impact of PBPK modeling in drug development and clinical practice.
(c) Raise awareness of the impact of pregnancy and age-related changes on drug disposition.
(d) Discuss key regulatory and clinical considerations when applying these models to inform drug dosing.

The hands-on sessions employed a PBPK platform technology (Simcyp ^®^ v21, Certara UK Limited, Simcyp Division, Sheffield, UK) to help attendees to

(a) Become familiar with basics of PK and PBPK modeling.
(b) Visualize the impact of anatomical and physiological changes on drug disposition.
(c) Understand the implications of dose modification in children and pregnant women.

Presentations of theoretical sessions were tailored according to the specific audience in a particular workshop and hands-on sessions were optimized with clinical cases that are generally familiar to the audience. The hands-on sessions were led by tutors in small groups each including 5 to 8 participants. Attendees accessed the Simcyp software via Amazon WorkSpaces. A mix of tutors from academia, industry or regulatory agencies enabled diverse discussions within each group. The technological requirement of a proprietary software such as Simcyp and the need to have tutors who are experienced users of the software for our workshops can be replicated using other PBPK software platforms, such as GastroPlus ^®^ ( https://www.simulations-plus.com/software/gastroplus/) and PKSim ^®^/Mobi ^®^ ( https://www.open-systems-pharmacology.org/). A hands-on exercise was followed by a plenary session, in which results and perspectives by each group were shared with other groups to stimulate interactive discussions. In one case study of a typical workshop (e.g., ESDPPP), delegates first simulated PK of anti-HIV drug efavirenz after a typical dose in the adult population, followed by simulating different doses in the pediatric population according to different pediatric dosing guidelines (e.g., those from US Food and Drug Administration and World Health Organization). The groups then explored the impact of either ethnicity, a weekends-off treatment regimen, or varying assumptions on CYP2B6 ontogeny on efavirenz pharmacokinetics. Results were presented in the plenary, and delegates reported their findings and related real life clinical experiences to the simulation results. 

Final agendas including speakers, presentations, and hands-on case studies for these five workshops can be found in Supplementary File 1.

## Effectiveness of our workshops

To understand the impact of our workshops, we conducted online surveys before and after the workshops (Supplementary file 2). Because the surveys are voluntary, not all participants responded ( Table 1). It was more challenging to obtain complete feedback after the workshop (for example, 36–75% of original responders before each workshop responded to the same question after the workshop at Radboud Summer School, ESDPPP and ASCPT events, Table 1), which limits the assessment of the impact of the workshop on changing attendees’ perspectives. As such, caution should be made when interpreting the survey results, especially when using percentage of responders to evaluate change in perspectives before and after the workshops. 

Some general findings from the surveys were:

- The majority of responders across all five workshops were considered ‘non-modelers’ because they were not familiar with PBPK (Figures S4–S6, S23, Supplementary file 2). For Radboud, ESDPPP, and ASCPT workshops, many of the respondents were PhD students, followed by postdoctoral researchers and pharmacists, mostly working in the field of pediatrics. Most delegates had only heard and read about PBPK modeling prior to the workshop, did not read PBPK modeling papers frequently, and had the impression that PBPK studies are difficult to interpret. Participants at the MHRA workshop were mostly pharmacists or clinicians working in obstetrics, and none of the responders were confident in PBPK before the workshop.
- The workshops appeared to be effective in leveling the ground for attendees to (i) understand state of science and technology and (ii) confidently apply PBPK. When asked ‘do you have sufficient knowledge and skills at this moment to conduct PBPK model simulations?’, most of the responders answered no before the workshops, yet they felt more capable of doing so after the workshop (Figure S7 of supplemental file 2). There was an increased percentage of responders after the workshop considering that ‘possessing mathematical and programming skills’ (Figure S10, supplemental file 2) is not necessarily essential. Similarly, responders at MHRA workshop tended to feel more confident in PBPK after the event (Figure S23–24, Supplemental file 2). The increased confidence and updated perspectives in attendees can be attributed to easy access to PBPK as a technology platform during the workshop, instead of modeling in the form of differential equations. With technology at hand, attendees can explore various modules constructed within the PBPK platform to visualize relevant physiology changes and pharmacological processes, execute simulations, and discuss simulation results within 30–45 minutes of a hands-on session. The experience of efficiently applying PBPK to answer clinical questions helped attendees to have updated views of PBPK.
- The attendees appeared to appreciate the value of PBPK in optimizing medicine use in under-served populations. For example, responders’ answer to the question 'are we at the point of using PBPK model output to inform drug labelling' tended to transition from being split to being confident that the field is ready to apply PBPK output to inform drug labels (Figure S15, Supplemental file 2). Responders also acknowledged the capability of PBPK to simulate drug exposure in fetuses upon maternal administration, as well as drug exposure in preterm and term neonates after the workshops (Figure S15, Supplemental file 2).
- All participants indicated the practical examples on dose adjustments with hands-on modeling as the most useful aspects. Many were interested in simulating dosing scenarios for specific drugs in tailored virtual patient populations, fostering follow-up discussions on research collaborations.
- Clinically oriented users asked for a simpler interface for the PBPK modeling software and expressed an interest in more real-world examples, particularly related to the top 10 frequently used medicines in clinical practice. Whilst some expressed the desire to have access to more user-friendly dosing tools based on contemporary PBPK technology, others however indicated that they still wanted to understand the science behind the software.

## Final thoughts

An adequate understanding of the concept and the state-of-the-technology of PBPK by healthcare providers is a critical step towards successful application of the tool to optimize pharmacotherapy in specific patient populations. Through these workshops, we effectively raised awareness of the relevance of PBPK in the context of clinical care, which reduces the barrier to implement PBPK modeling in informing drug dosing in children and pregnant women. We continue to collaborate in this space.

## Data Availability

Figshare. Supplemental files for "Demystifying Physiologically Based Pharmacokinetic Modeling among Non-Modelers towards Model-informed Medicine Use in Under-served Populations" (Submitted to Gates Open Research). DOI: 10.6084/m9.figshare.24153456 This project contains the following underlying data: Supplemental file 1 (Workshop agendas) Supplemental file 2 (survey results) Data are available under the terms of the Creative Commons Attribution 4.0 International license (CC-BY 4.0).
