# Supplementary material for: Demystifying physiologically based pharmacokinetic modelling among non-modelers towards model-informed medicine use in under-served populations
Source: Gates Open Res. 2023 Oct 26;7:128. [Version 1] doi: 10.12688/gatesopenres.14896.1 (PMC11259592; doi:10.12688/gatesopenres.14896.1)
Supplement: Peer review history

# Reviewer report: Reviewer response for version 1

**DOI:** 10.21956/gatesopenres.16225.r36314

**Published:** 2024-07-22

**Authors:** Kristina M. Brooks

**Affiliations:** 1 University of Colorado Anschutz Medical Campus, Aurora, USA

Freriksen et al. report on their experiences with providing workshops focused on educating non-modelers on the principles and potential applications of PBPK, with a more specific focus on pediatric, pregnant, and lactating populations. These educational initiatives are important for helping demonstrate the utility of this modeling approach to clinicians and researchers. General feedback from attendees appeared to confirm that these workshops did help “demystify” attendees’ understanding and ability to use/apply PBPK, and general findings from these trainings are helpful as a starting point for figuring out how to educate people on a larger scale.

 Please consider the following suggestions:

1. Pg. 4, Table 1: consider providing a breakdown of the amount of time spent listening to didactic sessions vs. hands-on learning activities alongside the total duration of the workshops
2. Pg. 4: The statement on “The technological requirement of a proprietary software…” felt a bit out of place and it did feel like some brief commentary on this aspect could also be helpful for readers. I would consider pulling this out into a separate paragraph/subheading on the ability to apply this to other software, but I do think explicitly noting open-source options and some of the pros/cons with this approach would be helpful if the authors are familiar with these other programs. Cost and ability to access software seem like important points to consider in addition to educating non-modelers in this space to ensure that these programs are used after the training.
3. Pg. 5: There is a statement on “We continue to collaborate in this space.” However, I do think some high-level commentary on future directions and ways to potentially increase access would be helpful to give readers a sense of how this educational space will continue to evolve.

Does the article adequately reference differing views and opinions?

Yes

Are all factual statements correct, and are statements and arguments made adequately supported by citations?

Yes

Is the Open Letter written in accessible language?

Yes

Where applicable, are recommendations and next steps explained clearly for others to follow?

Yes

Is the rationale for the Open Letter provided in sufficient detail?

Yes

Reviewer Expertise:

Clinical pharmacology, infectious diseases, pediatrics, pregnancy

I confirm that I have read this submission and believe that I have an appropriate level of expertise to confirm that it is of an acceptable scientific standard.

# Reviewer report: Reviewer response for version 1

**DOI:** 10.21956/gatesopenres.16225.r36765

**Published:** 2024-07-19

**Authors:** Ananth Kumar Kammala

**Affiliations:** 1 The University of Texas Medical Branch at Galveston, Texas, USA

The manuscript, “Demystifying physiologically based pharmacokinetic modelling among non-modelers towards model-informed medicine use in underserved populations,” highlights the success of workshops designed to demystify physiologically based pharmacokinetic (PBPK) modeling for non-modelers. These workshops have effectively raised awareness and understanding among clinicians, pharmacists, and academic researchers about using PBPK to optimize drug dosing in underserved populations.

 The workshops engaged diverse participants, including PhD students, postdoctoral researchers, pharmacists, and clinical assessors from regulatory agencies. This wide range of attendees indicates the broad relevance and applicability of PBPK modeling in different fields.

 Although it has several advantages but there are few limitations of the manuscript to be addressed.

1. The workshops relied on the proprietary software Simcyp®, which may not be accessible to all scientists. Although the manuscript suggests similar workshops could be conducted using other PBPK software platforms, such as GastroPlus® and PKSim®, the lack of direct experience with these alternatives is a limitation.
2. The manuscript notes that interpreting survey results, particularly when comparing percentages to absolute numbers, can be challenging. This complexity may introduce bias in understanding the true impact of the workshops.
3. By addressing these strengths and limitations, the manuscript provides a comprehensive overview of the value and challenges of using PBPK modeling to inform drug dosing in underserved populations.

Does the article adequately reference differing views and opinions?

Yes

Are all factual statements correct, and are statements and arguments made adequately supported by citations?

Yes

Is the Open Letter written in accessible language?

Yes

Where applicable, are recommendations and next steps explained clearly for others to follow?

Yes

Is the rationale for the Open Letter provided in sufficient detail?

Yes

Reviewer Expertise:

Reproductive pharmacology

I confirm that I have read this submission and believe that I have an appropriate level of expertise to confirm that it is of an acceptable scientific standard.

# Reviewer report: Reviewer response for version 1

**DOI:** 10.21956/gatesopenres.16225.r36766

**Published:** 2024-07-19

**Authors:** Anil Maharaj

**Affiliations:** 1 University of British Columbia, Vancouver, Canada

The submitted open letter summarizes 5 educational workshops on fostering an understanding of PBPK modeling to non-modelers and provides an analysis of feedback received from attendees. As PBPK modeling is commonly inferred to be an inherently complex process, the submitted letter, which details how educational workshops can be used to bridge the knowledge gap among non-modelers, is of contemporary interest. A key strength of this letter is the collection and analysis of quantitative survey data from workshop attendees as it serves to substantiate assertions/positions discussed by the authors. The following comments are focused on improving the clarity of the submitted letter:

 1.    Abstract: “As such, PBPK has the potential to enhance medicine use for populations that are often under-served globally in drug development and clinical care, namely pediatric patients, pregnant and lactating women.”

 Logical flaw - The opening sentences of the abstract do not provide an appropriate context/rationale as to why PBPK modeling is suitable for examining PK in underserved populations.

 2.    “To this end, computational tools such as physiologically based pharmacokinetic (PBPK) modeling which characterize the physiological changes can be used to support dosing decisions (3–5)”

 References (particularly ref 4 and 5) do not discuss the use of PBPK modeling to support drug dosing decisions.

 3.    “In parallel, one can compile drug-dependent parameters to construct a drug model, and couple it with the system model through relevant physiological processes concerning the absorption and dis- position of the drug.”

 Confusing sentence – unclear as to what is the output from combining drug- and system-specific parameters. Consider revising to indicate that combining drug- and system-specific parameters allows models to generate PK estimates that are developmentally specific.

 4.    Suggest defining the acronym MHRA in the body of the letter. 

 5.    Suggest indicating how many (of the 5) workshops were online and how many were in-person within the body of the letter.

 6.    Why were MHRA survey results not integrated with the rest of the workshops in supplementary document 2? Were the survey questions for this workshop unique or different?

 7.    Consider including dialogue on how future workshops could be improved (or separating the ‘Effectiveness of our workshops’ section between strengths and areas for improvement).

 8.    “The increased confidence and updated perspectives in attendees can be attributed to easy access to PBPK as a technology platform during the workshop, instead of modeling in the form of differential equations.”

 Do survey results directly support this statement? If so, please include dialogue that links this proposition to quantitative results from the survey.

Does the article adequately reference differing views and opinions?

Yes

Are all factual statements correct, and are statements and arguments made adequately supported by citations?

Yes

Is the Open Letter written in accessible language?

Yes

Where applicable, are recommendations and next steps explained clearly for others to follow?

Yes

Is the rationale for the Open Letter provided in sufficient detail?

Yes

Reviewer Expertise:

Pharmacokinetic modeling, pediatrics, pregnancy, and lactation.

I confirm that I have read this submission and believe that I have an appropriate level of expertise to confirm that it is of an acceptable scientific standard, however I have significant reservations, as outlined above.

# Reviewer report: Reviewer response for version 1

**DOI:** 10.21956/gatesopenres.16225.r36760

**Published:** 2024-07-19

**Authors:** Li Guo-Fu

**Affiliations:** 1 China Pharmaceutical University,, Nanjing, China; 2 Subei People's Hospital, Yangzhou, China

I find this open letter written by renowned and experienced experts in the field of PBPK modeling to be both interesting and informative. I have one suggestion: should the authors emphasize the best practice strategy in the use of PBPK modeling to address specific medicine use questions in underserved populations? Highlighting the best practice, particularly regarding the differing levels of quantification or verification of PBPK, could enhance the confidence of non-modeler attendees in using their PBPK models to address their intended questions.

Does the article adequately reference differing views and opinions?

Yes

Are all factual statements correct, and are statements and arguments made adequately supported by citations?

Yes

Is the Open Letter written in accessible language?

Yes

Where applicable, are recommendations and next steps explained clearly for others to follow?

Yes

Is the rationale for the Open Letter provided in sufficient detail?

Yes

Reviewer Expertise:

Physiologically-based pharmacokinetic modeling; clinical pharmacology; clinical pharmacy; biostatistics

I confirm that I have read this submission and believe that I have an appropriate level of expertise to confirm that it is of an acceptable scientific standard.

# Reviewer report: Reviewer response for version 1

**DOI:** 10.21956/gatesopenres.16225.r36318

**Published:** 2024-07-19

**Authors:** Ogochukwu Ukamaka Amaeze

**Affiliations:** 1 University of Washington, Washington, USA; 2 Clinical Pharmacy and Biopharmacy, University of Lagos, Yaba, Federal Capital Territory, Nigeria

The paper “Demystifying physiologically based pharmacokinetic modelling among non-modelers towards model-informed medicine use in under-served populations” outlines the details and outcomes of five workshops designed for non-modelers. These workshops aimed to impart knowledge of the science and technology behind PBPK modeling and foster confidence in its application to enhance drug therapy in underserved populations, including children and pregnant women. The paper highlights the benefits of the workshops in equipping participants with the necessary understanding and confidence to potentially harness PBPK modeling effectively in clinical practice.

 Please find my comments below for the author’s consideration.

1. While the abstract effectively defines under-served populations, the paper omits a definition of the term “non-modelers”. It would be beneficial for the authors to incorporate a clear explanation of what constitutes a non-modeler in the second section, titled "Demystifying PBPK among non-modelers for wider use in under-served populations." This addition will provide valuable context for the workshop target audience - non-modelers.
2. Page 3 - In the statement, "The workshops appeared to be effective in leveling the ground for attendees to (i) understand the state of science and technology and (ii) confidently apply PBPK," consider adding "PBPK" before "science" in (i) to specify the domain. Additionally, for (ii), consider expanding on the applications, such as applying PBPK in clinical practice, research, and related contexts.
3. It would be helpful for the authors to include a discussion on the challenges encountered during the workshops and any inherent limitations. Drawing from their experiences and insights from organizing these workshops, offering recommendations for effectively organizing similar events worldwide would be invaluable. These recommendations could encompass considerations such as the mode of delivery (online versus in-person), optimal duration, software technologies, and other pertinent factors. This guidance would assist individuals interested in organizing similar workshops in the future.
4. A minor point – the text in the final row and column in the table is missing a closing bracket.

Does the article adequately reference differing views and opinions?

Yes

Are all factual statements correct, and are statements and arguments made adequately supported by citations?

Yes

Is the Open Letter written in accessible language?

Yes

Where applicable, are recommendations and next steps explained clearly for others to follow?

Partly

Is the rationale for the Open Letter provided in sufficient detail?

Yes

Reviewer Expertise:

Clinical Pharmacology, Maternal and Pediatric Pharmacology, PBPK Modeling.

I confirm that I have read this submission and believe that I have an appropriate level of expertise to confirm that it is of an acceptable scientific standard, however I have significant reservations, as outlined above.

# Reviewer report: Reviewer response for version 1

**DOI:** 10.21956/gatesopenres.16225.r35428

**Published:** 2023-11-14

**Authors:** Eric Chan

**Affiliations:** 1 National University of Singapore, Singapore, Singapore

The reviewer concurs that an understanding of the concepts of PBPK by healthcare providers is crucial for the successful application of PBPK modeling to optimize pharmacotherapy in specific patient populations. Through the workshops, the authors effectively raised awareness of the relevance of PBPK in optimizing clinical pharmacotherapy. The workshops also help to reduce the barrier clinical practitioners in implementing PBPK modeling to informing drug dosing in children and pregnant women. Similar workshops should be conducted in other parts of the world.

 Below are comments for the authors to consider.

1. Abstract. It is stated that ‘As such, PBPK has the potential to enhance medicine use for populations that are often under-served globally in drug development and clinical care, namely pediatric patients, pregnant and lactating women’. Perhaps the authors should consider including geriatric patients in this statement even though their focus is on pediatric and obstetric pharmacology subsequently. As geriatric patients are often excluded from clinical trials just like the pediatric patients, pregnant and lactating women, citing them may create the awareness of urgent work needed in the geriatric areas as well.
2. Page 4. It is mentioned that ‘The technological requirement of a proprietary software such as Simcyp and the need to have tutors who are experienced users of the software for our workshops can be replicated using other PBPK software platforms, such as GastroPlus ® ( https://www.simulations-plus.com/software/gastroplus/) and PKSim®/Mobi® ( https://www.open-systems-pharmacology.org/)’. The reviewer is wondering if such alternative software platforms have been tested before in similar workshops conducted by the authors? If yes, sharing their experiences in this paper briefly may be important as not all scientists have access to the Simcyp software.
3. Using Figure S12 as an example, it is relatively difficult to interpret the bar graph. For example, under ‘no’ as a response, the percentage before workshop was about 10% while the percentage after the workshop was about 20% (2-fold higher). But if we examine the absolute number of responders, they are comparable. The reviewer understands the nuances between percentage versus absolute number, but the interpretation of the results can be biased depending on which read-outs the authors select to present and discuss. Please clarify.
4. The paper discusses many benefits of the workshops. Are there any limitations, challenges and considerations? Please discuss.

Does the article adequately reference differing views and opinions?

Yes

Are all factual statements correct, and are statements and arguments made adequately supported by citations?

Yes

Is the Open Letter written in accessible language?

Yes

Where applicable, are recommendations and next steps explained clearly for others to follow?

Yes

Is the rationale for the Open Letter provided in sufficient detail?

Yes

Reviewer Expertise:

Physiologically based pharmacokinetic modeling

I confirm that I have read this submission and believe that I have an appropriate level of expertise to confirm that it is of an acceptable scientific standard.

# Response

**Published:** 2023-12-16

**Authors:** Ping Zhao

**Affiliations:** Integrated Development, Bill & Melinda Gates Foundation, Seattle, Washington, USA

The authors greatly appreciate comments provided by this reviewer.  Below are the responses to reviewer's comments:

 1. Abstract. It is stated that ‘As such, PBPK has the potential to enhance medicine use for populations that are often under-served globally in drug development and clinical care, namely pediatric patients, pregnant and lactating women’. Perhaps the authors should consider including geriatric patients in this statement even though their focus is on pediatric and obstetric pharmacology subsequently. As geriatric patients are often excluded from clinical trials just like the pediatric patients, pregnant and lactating women, citing them may create the awareness of urgent work needed in the geriatric areas as well.

*Response: We agree and will add the special population ‘geriatric patients’ to this list, with the notification that this specific population was not included in our workshops.*

 2. Page 4. It is mentioned that ‘The technological requirement of a proprietary software such as Simcyp and the need to have tutors who are experienced users of the software for our workshops can be replicated using other PBPK software platforms, such as GastroPlus ® (https://www.simulations-plus.com/software/gastroplus/) and PKSim®/Mobi® (https://www.open-systems-pharmacology.org/)’. The reviewer is wondering if such alternative software platforms have been tested before in similar workshops conducted by the authors? If yes, sharing their experiences in this paper briefly may be important as not all scientists have access to the Simcyp software.

*Response: No, we do not have experience with workshops using other software, we merely suggest that these workshops can also be conducted with other software platforms like PK-Sim and Gastroplus.*

 Using Figure S12 as an example, it is relatively difficult to interpret the bar graph. For example, under ‘no’ as a response, the percentage before workshop was about 10% while the percentage after the workshop was about 20% (2-fold higher). But if we examine the absolute number of responders, they are comparable. The reviewer understands the nuances between percentage versus absolute number, but the interpretation of the results can be biased depending on which read-outs the authors select to present and discuss. Please clarify.

*Response: We appreciate that the reviewer addresses this. We had already the discussion internally on how to present the data in the supplementary file as we felt that both were important, i.e., percentages as well as absolute numbers, as the absolute numbers differ between the before and after workshops while percentages are more easier for interpretation. Hence, we decided to present both in the supplementary materials to be as comprehensive as possible and unbiased.*

 The paper discusses many benefits of the workshops. Are there any limitations, challenges and considerations? Please discuss.

*Response: There indeed are limitations and challenges with the workshops as well. We now discuss some limitations and challenges as well, see the addition below.*

*‘Limitations and challenges of the workshops*

*While the workshops have demonstrated significant value, we acknowledge certain limitations. First, these events have been conducted either in person or online, each possessing its own set of advantages and drawbacks. Online workshops, for instance, are more time-efficient, globally accessible, and cost-effective due to the elimination of travel requirements. However, in-person workshops foster more interactive discussions among delegates, albeit posing challenges in reaching stakeholders on a larger global scale.  Second, the workshops have primarily focused on infectious diseases affecting children and pregnant women in low and middle-income countries. While this focus has been beneficial, recent advancements in PBPK offer a broader learning experience. Consequently, discussions regarding the successes and challenges of applying PBPK to support dosing in other underserved populations, such as lactating women and geriatric populations, should be integrated into these workshops.'*
